# Does doctor know best? The recent trend in medical negligence

**DOI:** 10.2349/biij.5.1.e12

**Published:** 2009-01-01

**Authors:** FS Shuaib, IL Shuaib

**Affiliations:** 1 Ahmad Ibrahim Kulliyyah of Laws, International Islamic University Malaysia, Kuala Lumpur, Malaysia; 2 Institut Perubatan dan Pergigian Termaju, Universiti Sains Malaysia, Pulau Pinang, Malaysia

## INTRODUCTION

Presently, the tort system is used to regulate medical negligence litigation in Malaysia. Generally, this system provides for compensation only when a doctor or any other medical personnel assisting in the treatment of a patient is negligent. Previously, in determining whether a doctor was negligent in diagnosis, treatment and advice, the court had shown a deferential attitude towards medical judgment. This is in contrast to the attitude of the court towards other professions such as engineers and architectures where the court does not hesitate in questioning the appropriateness and reasoning of the standard practice adopted by those professionals. However, this deferential attitude which is encapsulated in the phrase “a doctor knows best” is slowly dissipating.

This article will look at this development of the law by highlighting selected landmark cases that enumerate this change in court’s attitude [1]. It is important for radiologists, in Malaysia in particular, to understand the implication in everyday practice.

## *BOLAM V FRIERN HOSPITAL*(1957):A DOCTOR KNOWS BESTd

During this period, the general view was that the doctor knows best and even judges should not question the doctor’s opinions. The test of determining negligence in *Bolam*’s case was not to state doctors could not be negligent, but if the doctor had followed one of the responsible divergent opinions, he could not be faulted. Judges were not at liberty to question the validity or appropriateness of the opinion followed. In other words, the negligence of a medical practice should be determined by fellow medical practitioners, not judges.

In the case of *Bolam v Friern Hospital Management Committee* [2], a voluntary psychiatric patient at Friern Hospital suffered bilateral “stove-in” fractures of the acetabula during a course of electro-convulsive therapy (ECT) treatment administered to him in August 1954. The ECT treatment was administered without a relaxant drug or any form of manual restraint other than to support the plaintiff's chin and hold his shoulders. Nurses were present on either side of the couch in case the plaintiff fell off. The plaintiff claimed damages, alleging that the doctor was negligent in failing to administer any relaxant drug or to provide at least some form of manual restraint and in failing to warn him of the 1:10,000 risk of fracture associated with the treatment.

Although the doctor admitted that he knew that some doctors adopted other procedures such as providing a relaxant drug to patients, his training informed him otherwise. The patient produced an expert witness – a distinguished psychiatrist – who described the failure to administer manual control over the patient as foolhardy. It was also the expert witness’ standard practice to warn patients of the relevant risks of ECT. The hospital involved, on the other hand, produced other expert witnesses who concurred with the procedure adopted by the defending doctor and who considered informing the patient of risk of fracture as unnecessary. Since the practice of doctors was supported by a body of medical opinions, the standard of the defending doctor’s practice could not be questioned and as such the doctor was acquitted by the jury.

The principle enunciated in *Bolam*’s case discourages second guessing any medical judgment even by fellow doctors. Thus, as long as the practice of a doctor is supported by a body of medical opinion, it is not the business of the court to question the appropriateness of that body of opinion. Perhaps such judicial attitudes could be explained by the general attitude of the time where paternalism in all forms was the norm. Those in authority or possessing repository of professional knowledge should be given the privilege to decide for others. This paradigm was set to change.

## *ROGERS V WHITAKER* (1992): A PATIENT CAN DECIDE FOR HIMSELF

*Rogers v Whitaker* [3], an Australian case, has been widely earmarked as a departure point in which the blatant paternalism in the previous era was jettisoned. Rather than allowing medical opinion to prevail even on patients’ decision making, the court is willing to re-examine the appropriateness of the standard adopted by doctors.

In this case, Maree Lynette Whitaker consulted an ophthalmic surgeon regarding her right eye, which was becoming almost blind. The surgeon advised her that an operation on that eye would probably restore significant sight to it. She agreed to undergo the operation, but unfortunately the operation did not improve her sight. Unfortunately, she developed sympathetic ophthalmia in her left eye (a recognised risk of 1 in 14,000), which caused her to lose all sight in the left eye. There was no allegation of negligent in the performance of the surgery itself. What was in question was the failure of the surgeon to inform her of the danger of sympathetic ophthalmia in her other eye. The Court found the surgeon to be negligent in failing to inform her of the said risk, despite the incessant inquiries of the patient on any side-effects of the surgery over her “good” eye.

For the surgeon, the risk of sympathetic ophthalmia “was not something that came to [his] mind to mention to her” [3]. Expert opinions were produced both supporting and against the practice of informing of such risk. However, had the court decided to follow *Bolam’s* case, the court could not evaluate the contradictory medical opinions and thus a doctor would not have been negligent as long as his practice is in accord with at least one body of medical opinion.

The Australian court stressed the importance of autonomous decision making of patients. In this regard, perhaps the human rights discourse of self determination and autonomy of an individual had some influence in this shift. Thus, the court has the ultimate responsibility to determine whether a practice conforms to the standard of reasonable care demanded by law. More importantly, this responsibility could not be delegated to the profession.

## *BOLITHO V CITY AND HACKNEY HEALTH AUTHORITY* (1997): JUDGES ALSO CAN THINK

The weight given in *Rogers v Whitaker* where medical profession should not have the final say in determining the standard of reasonable care was repeated in *Bolitho*’s case.

In this case [4], a two-year-old patient suffered brain damage as a result of cardiac arrest induced by respiratory failure. He was admitted for croup and had episodes of breathing difficulty during his stay at the St. Bartholomew's Hospital. In spite of calls made by the nurses to doctors regarding the patient’s breathing difficulty, none came. One of the questions that the court had to answer was: “Had the doctors come, should the doctors have intubated the patient which could have saved him?” The expert witnesses called to testify provided conflicting opinions. The trial judge surmised that even if the view not to intubate was unreasonable and illogical, she could not substitute her own views for those of the medical experts. This is in line with the *Bolam*’s test where a doctor is not negligent as long as there is a body of opinion that supported his practice.

However, the House of Lords, the court of final appeal in England, disagreed with the reservations of the trial judge to substitute her opinion for those of the medical experts. The opinion of the House of Lords that the court can evaluate has logical basis of medical opinions. In weighing risks against benefits, a judge must be satisfied that medical experts “have directed their minds to the question of comparative risks and benefits and have reached a defensible conclusion.” [4]

*Bolitho*’s case made it clear that a judge could pierce through the medical opinions and determine the reasoning of such opinions. Although in most cases, “distinguished experts in the field are of a particular opinion and will demonstrate the reasoning of that opinion.” However, similar to standard of care for other professions, the court now has the ultimate responsibility to determine the reasoning of such standard.

## *FOO FIO NA V DR. SOO FOOK MUN*(2007): THE NEW MILLENNIUM APPROACH

This trend in departing away from medicalism is followed in Malaysia in the case of *Foo Fio Na v Dr. Soo Fook Mun* [5]. Fifty years after *Bolam*’s case, the Federal Court, the final court of appeal in Malaysia, decided that indeed judges could disagree with medical opinions.

The patient in this case suffered closed dislocation C4 and C5 vertebrae with bilaterally locket facets after being involved in a car accident. An orthopaedic surgeon performed a surgery where the dislocated vertebrae were moved to their normal positions and secured by bone grafting and insertion of a loop of wire. The wire loop was found to cause total paralysis of the patient by pressing on the spinal cord. Although the patient signed a general consent form during admission, the patient claimed that she was not informed of the risk of paralysis from the particular surgery. The court found that the doctor was negligent in failing to inform her of the risk.

The Court viewed the *Bolam*’s approach as being “over protective and deferential” to the medical profession [5]. It is the court that determines reasoning of doctors’ conduct, and not the profession. The Federal Court opined that “the *Rogers v Whitaker* test would be a more appropriate and a viable test of this millennium.” [5]

## CONCLUSION AND GENERAL PRINCIPLES

The four cases cited above show that there is a shift in determining a reasonable standard of medical care. The deferential approach in yesteryears is taken over by a neutral, albeit still tentative, approach as in other professions. Below, are some principles that can be gleaned from the cases.

On setting the standard of reasonable care, the recent cases have made it clear that the court could substitute its judgment for those of medical experts if such expert opinions fail under the court’s logical analysis. Thus, simply producing an expert opinion that agrees with the practice in question may not be enough. However, *Bolitho*’s case reminded everyone that the court will not be hasty in challenging the opinions of distinguished experts. Only in rare cases that the court may have to assert that it is the court that has the ultimate responsibility in determining a reasonable standard.

Secondly, the principle of informed consent is here in Malaysia. A general consent form is meaningless if the patient is not informed of relevant risks of the procedure. For example, a radiologist not informing the patient the possibility of developing an anaphylactic shock after iodine contrast intravenous injection [6]. The important consideration is on the ability of the patient to make his own decision after receiving relevant information from the doctors. Whether a particular information is relevant depends also from the point of view of the patient, and not necessarily the opinions of the doctors.

Thirdly, the court may no longer give the benefit of doubt to doctors in cases of missing X-ray films. The court will make adverse inference as allowed by law if doctors or hospitals fail to produce X-ray films or documents in the court [7]. As in other cases, the court may make adverse inference against doctors.

Fourthly, the court subscribed to prevailing perception that compared with other professions that the medical profession in some instances failed “to stand up to the wrong doings” of their brethren [5]. The impact of the effect of this perception to the approach taken by the court is not clear. Perhaps it may lend credence to the need for the court to form its own opinion about a reasonable standard of care in diagnosis, treatment and advice.

Thus, although there were differences of expert opinions with regard to a reasonable decision of a vascular interventional radiologist to proceed with a renal angioplasty after being aware of an anatomical variation, a court found that the radiologist had taken all the reasonable steps necessary to ensure proper placement of the balloon [8].
